# Muscular performance analysis in “cross” modalities: comparison between “AMRAP,” “EMOM” and “RFT” configurations

**DOI:** 10.3389/fphys.2024.1358191

**Published:** 2024-03-05

**Authors:** Manuel Barba-Ruíz, Francisco Hermosilla-Perona, Juan Ramon Heredia-Elvar, Noelia Gómez-González, Marzo Edir Da Silva-Grigoletto, Diego Muriarte-Solana

**Affiliations:** ^1^ Department of Physical Activity and Sports Science, Alfonso X El Sabio University, Madrid, Spain; ^2^ Facultad de Ciencias de la Vida y la Naturaleza, Universidad Nebrija, Madrid, Spain; ^3^ Department of Physical Education, Federal University of Sergipe, São Cristóvão, Brazil

**Keywords:** training, strength, squat, pacing, fatigue, cross, iGEM

## Abstract

**Introduction:** In recent years, a surge of interest in high-intensity training methods, associated with “cross” modalities has emerged as a promising approach for improving performance and overall health. Therefore, the main aim of this study was to compare the acute effects on heart rate, mean propulsive velocity and intra and inter-set velocity loss in “Cross” modalities.

**Materials and methods:** Twelve athletes, 10 men’s and 2 women’s (age: 31.5 ± 6.74 years; height: 174.17 ± 6.05 cm; weight: 75.34 ± 7.16 kg) with at least 1 year of experience in “cross” training. The participants performed three different “cross” modalities, Rounds for Time (RFT), Every Minute on the Minute (EMOM) and As Many Rounds As Possible (AMRAP) across three separate days. In each modality participants carried out 10 repetitions of squat, pull-ups, and shoulder press with difference rates of work-rest. Mean propulsive velocity (MPV) and heart rate (HR) were recorded and analysed for each athlete. Repeated measures one-way ANOVA and repeated measures two-way ANOVA were performed to analyse the differences between modalities and subjects. Besides, a Bonferroni *post hoc* analysis was carried out to assess the differences between modalities in each subject.

**Results:** Significant differences in MPV were observed among the modalities. The comparisons between RFT and AMRAP, as well as EMOM and AMRAP, revealed lower MPV in the AMRAP modality (*p* < 0.01). RFT exhibited the greatest intra-set velocity loss, while EMOM showed the least, with significant distinctions (*p* < 0.01) between them. Furthermore, significant differences in the HR results were noted among all modalities (*p* < 0.05).

**Conclusion:** Findings consistently identify the AMRAP modality as having the lowest MPV values due to its prolonged duration, promoting self-regulated tempo for optimal performance and technique, while the RFT modality exhibits higher fatigue and intra-set MPV losses. These insights into propulsive velocity, intensity, fatigue, and pacing across various “Cross” modalities provide valuable guidance for athletes and trainers seeking to enhance their exercise programs.

## 1 Introduction

In recent years, there has been a growing interest in “high-intensity” training as a viable alternative for enhancing both performance and overall health (Meyer et al., 2017; Claudino et al., 2018; Feito et al., 2018). These training methods have given rise to various modalities, initially linked with the CrossFit^®^ brand (Glassman, 2002; Smith et al., 2013; Meyer et al., 2017; Claudino et al., 2018; Feito. et al., 2018), often referred to by different names such as Functional Fitness, Extreme Conditioning Programs, Mixed Modality Training, High-Intensity Functional Training, and Cross Training, which have collectively been categorized as “cross” modalities (De-Oliveira et al., 2021).Initially, these “cross” modalities were designed to offer high-intensity training regimens. These regimens encompass a fusion of exercises drawn from weightlifting and other strength-related activities, such as pull-ups, weighted exercises, and deadlifts, alongside gymnastic and calisthenic movements like pull-ups, muscle-ups, and handstands, in addition to resistance exercises like sprints, rowing, and rope jumping. These exercises are categorised according to the Gymnastic-Metabolic-Weightlifting (G-M-W) model, aiming to create workout configurations characterised by their intensity and a considerable degree of exercise variety (Maté-Muñoz et al., 2018). In essence, these workouts are centered on incorporating movements that are either more or less intricate, involve multiple joints, and exhibit diversity. These movements are executed with brief recovery intervals between them (Drum et al., 2017; Maté-Muñoz et al., 2018).Typically, these training sessions are structured to prioritise the central component known as the “Workouts of the Day” (WOD) (Glassman, 2002).

The formulation of these WODs generally adheres to one of two key principles: either they are established based on predetermined time durations, or they are defined by specific tasks to be accomplished. In the latter scenario, the task-driven criteria influence not only the nature of the exercises but also the time required for their successful completion (De-Oliveira et al., 2021). In this sense, various training methodologies have been devised, comprising the AMRAP (“As Many Repetitions/Rounds As Possible”) approach, where the objective is to complete the maximum number of specified repetitions or rounds within a prescribed time limit over periods of 10–20 min. Another modality is RFT (“Rounds For Time”), where the goal is to accomplish the designated number of rounds in the briefest amount of time possible. Lastly, the EMOM (“Every Minute on the Minute”) method involves performing the specified repetitions within a 1-min interval, with work and rest periods occurring within each minute (Smith et al., 2013; Drum et al., 2017; Maté-Muñoz et al., 2018; Silva-Grigoletto et al., 2020; De-Oliveira et al., 2021).

Within the realm of research, there exist inquiries aimed at scrutinising the immediate impacts of these training configurations (Gómez-Landero and Frías-Menacho, 2020), with a particular emphasis on factors such as fatigue levels associated with these methodologies (De-Oliveira et al., 2022). Despite the difficulty in defining and controlling the training stimulus, given the variable characteristics of the programs (Mangine and Seay, 2022), we will focus on the strength exercises that are usually part of these programs. In this regard, the vast majority of the studies analyse the effects on similar absolute loads or, failing that, relative loads established with respect to the value of 1RM (Maté-Muñoz et al., 2018; Silva-Grigoletto et al., 2020; De-Oliveira et al., 2021; De-Oliveira et al., 2022), which can present serious limitations (Sanchez-Medina and González-Badillo, 2011; Elvar-Heredia et al., 2021; Maté-Muñoz et al., 2022). Thus, propulsive velocity has been identified as the most precise variable for evaluating the intensity of strength exercises (Sanchez-Medina et al., 2010; Sanchez-Medina and González-Badillo, 2011). Accordingly, athletes who attain higher mean propulsive velocity (MPV) at the same load exhibit lower relative intensity values.

Muscle fatigue is recognized as a complex, task-dependent and multifactorial phenomenon (Enoka and Stuart, 1992; Enoka and Duchateau, 2008; Place et al., 2010) however, it is acceptable by the scientific community defined as an exercise-induced transient decline in muscle force-generating capacity (Enoka and Stuart, 1992; Allen et al., 2008; Enoka and Duchateau, 2008). During typical resistance exercise in isoinertial conditions, and assuming every repetition is performed with maximal voluntary effort, velocity unintentionally declines as fatigue develops (Izquierdo et al., 2006). In this sense, MPV loss across the repetitions is considered a good muscular fatigue indicator in isoinertial exercises (Sanchez-Medina and González-Badillo, 2011). A further issue concerning this variable and their regulation is that individuals engaging in these practices may employ specific strategies to autonomously regulate their performance levels and recuperation across diverse configurations to manage fatigue levels (Gómez-Landero and Frías-Menacho, 2020; De-Oliveira et al., 2021). This fact presents significant heterogeneity and challenges in the realm of regulating the intensity variables and intra-session recovery (De-Oliveira et al., 2021; Mangine et al., 2021).

These constraints collectively hinder our ability to achieve a more accurate comprehension of the specific mechanical and metabolic stimuli inherent in such training configurations. In these programs, it is worth noting that while the relative intensity is often not exceedingly high or at maximum levels, the methodology is structured around temporal fluctuations concerning the stimulus and recovery. Consequently, it is necessary to deepen the analysis of the performance demands associated with each configuration and to empower exercise professionals to formulate design decisions founded on criterion rooted in objective data. In this sense, the main aim of this study was to compare the acute effects on heart rate, mean propulsive velocity and intra and inter-set velocity loss in “Cross” modalities (AMRAP, RFT and EMOM). It would be hypothesise that EMOM, with have mandatory rests, likely results in lower heart rate values and higher MPV compared to other routines. However, the volume differences effects on heart rate and MPV during routines without rest, such as RFT and AMRAP, are not clearly understood.

## 2 Materials and methods

### 2.1 Participants

A descriptive experimental cross-sectional study was carried out, involving a sample of 12 participants (age: 31.5 ± 6.74 years; height: 174.17 ± 6.05 cm; weight: 75.34 ± 7.16 kg), 10 men’s and 2 women’s, all of whom have a minimum of 1 year of experience in “cross” training. The selected participants in this study had not reported any injuries during the study period nor in the 6 months leading up to the commencement of the research.

The experimental procedures were thoroughly explained to the participants, who were provided with information regarding the potential risks associated with the experiments. Prior to their involvement in the study, all participants gave their informed consent in writing.

### 2.2 Procedure

The participants engaged in three different “cross” modalities, Rounds for Time (RFT), Every Minute on the Minute (EMOM) and As Many Rounds As Possible (AMRAP) across three separate days, allowing for a full week of recovery between sessions. All measurements took place between approximately 8:30 a.m. and 12:00 p.m. in similar ambient conditions (15ºC–20°C and 30%–40% humidity).

In each of these exercise programs, three specific exercises were performed consistently: squat, pull-ups, and shoulder press. It is important to note that the absolute loads and repetitions for these exercises remained identical across all the programs. However, the number of sets and rest periods varied depending on the unique characteristics of each modality. During the AMRAP, participants were instructed to complete 10 repetitions of each exercise and maximise the number of sets and rounds within a 12-min timeframe. In the course of this program, participants were encouraged to set their own pace, with the primary goal being to achieve as many rounds as possible. In the EMOM, each exercise was performed within a 1-min interval, comprising both the work and rest periods. Participants completed 10 repetitions of each exercise within this time frame, then rested until the minute before moving on to the next exercise. Finally, in RFT, participants were directed to complete half of the rounds they had previously achieved in the AMRAP but in the shortest time possible.

For the collection of heart rate data (HR), a heart rate monitor (Wahoo Tickr) was affixed to the chest using an elastic band. Such data was recorded every 30 s. Mean propulsive velocity (MPV) was registered during the squat exercise at both the initial and final repetitions of each squat set using an optoelectrical encoder (Velowin).

### 2.3 Statistical analysis

The mean ± standard deviation (SD) was obtained for descriptive analysis of the study variables. To ensure the reliability of subsequent statistical analyses, the normality of the data distribution was assessed and confirmed through the Shapiro-Wilks test.

A repeated measures one-way ANOVA was performed to analyse the differences between modalities and the variables of heart rate, MPV, and intra- and inter-set loss velocity. A two-way repeated measures ANOVA was performed to analyse the differences in MPV between each of the subjects and the modality (subject*modality). A Bonferroni *post hoc* analysis was conducted to assess the differences between modalities in each participant.

All analyses were conducted using SPSS v. 24.0 statistical software for Mac Os (IBM SPSS Statistics) and GraphPad Prism 8 software. The significance level was set at *p* < 0.05.

## 3 Results

**FIGURE 1:**
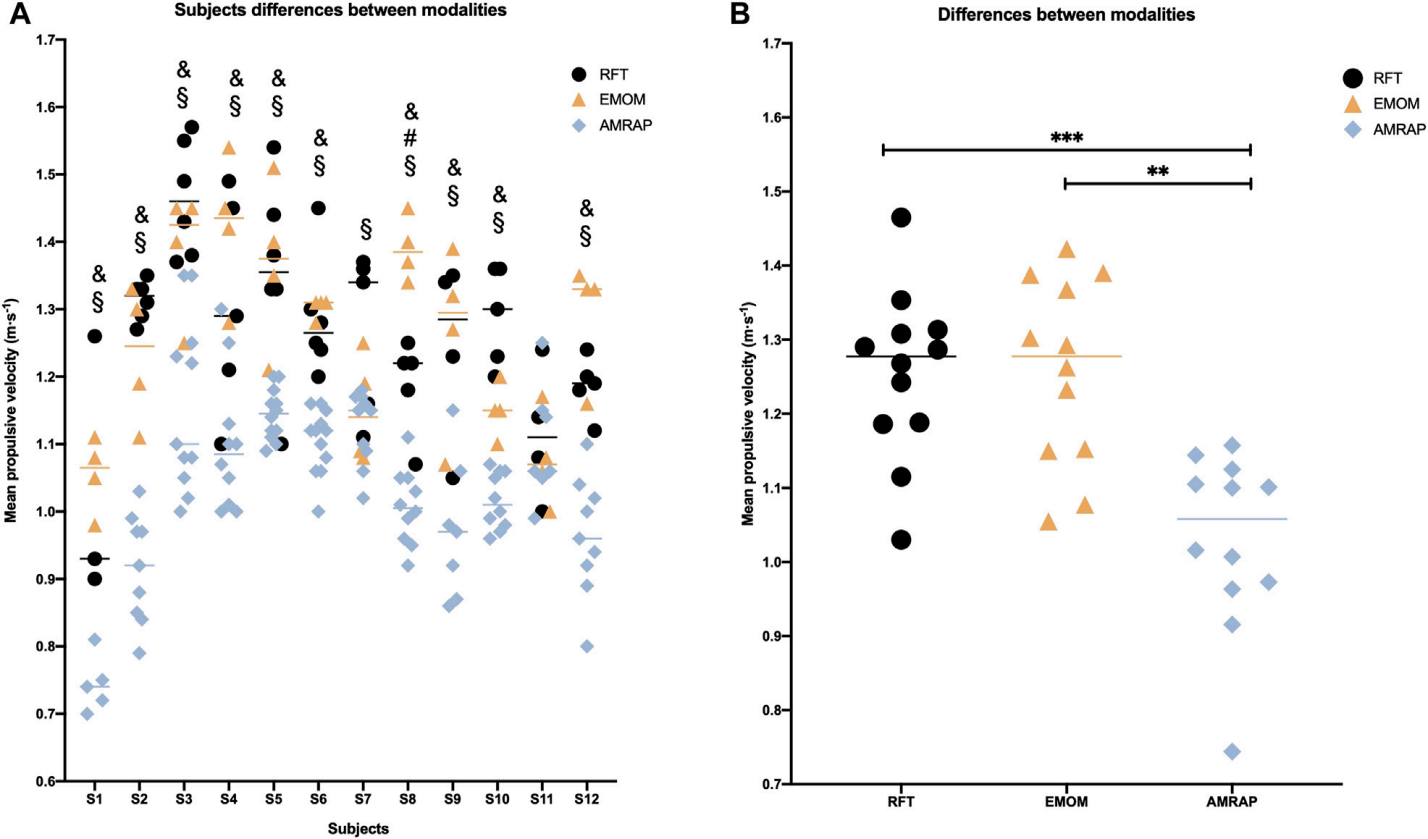
Differences in the velocity of the first repetition of each series according to the modalities **(B)** and the subjects **(A)** Note: # Significant differences for α < 0.05 between RFT and EMOM; § Significant differences for α < 0.05 between RFT and AMRAP; and Significant differences for α < 0.05 between EMOM and AMRAP; *** Significant differences for α < 0.001; ** Significant differences for α < 0.01; * Significant differences for α < 0.05.

The results according to the variations in MPV between modalities in each subject showed significant differences between RFT and AMRAP (*p* < 0.05) and between EMOM and AMRAP (*p* < 0.05) except for subject 11, with always lower MPV in AMRAP (Figure 1A). In the case of the analysis of the MPV between modalities, there can be observed significant differences (*p* < 0.001) between RFT and AMRAP and between EMOM and AMRAP (*p* < 0.01) with lower speeds for the AMRAP. However, no significant differences in MPV were obtained between RFT and EMOM modalities (Figure 1B).

**FIGURE 2:**
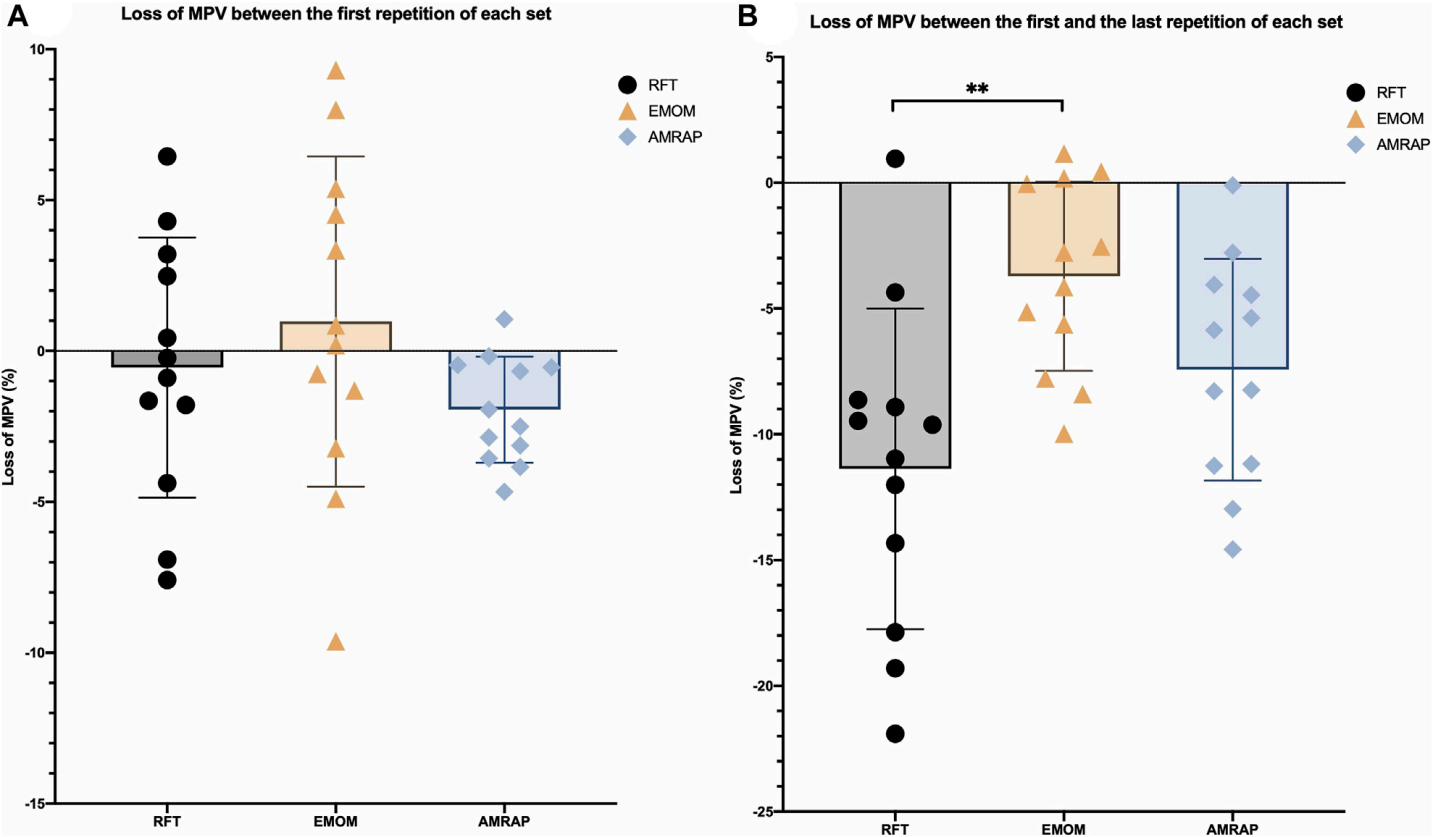
Percentage loss of velocity between the first repetition of subsequent sets **(A)** and between the first and last repetition of each set **(B)**. Note: MVP: mean propulsive velocity; * Significant differences for α < 0.05, ** Significant differences for α < 0.01.

The loss of MPV amongst modalities between the first repetition of each set does not present statistical differences (Figure 2A). However, in the EMOM, the results showed a gain in MPV throughout the sets. Regarding to the analysis between the first and the last repetition of each set (Figure 2B), RFT showed the greatest loss of MPV with statistical differences from EMOM (*p* < 0.01). Nevertheless, AMRAP does not present any statistical differences from the rest of the modalities.

**FIGURE 3:**
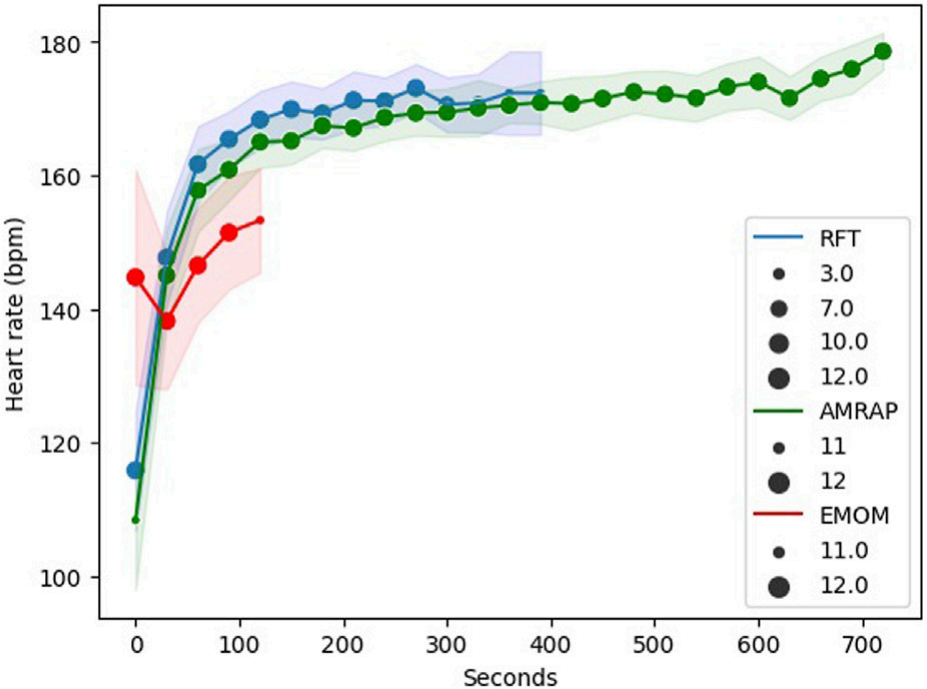
Descriptive Heart Rate (HR) data in each modality. Note: dots represent the mean and shaded area the standard deviation.

Descriptive HR data (Figure 3) showed that, while AMRAP yields the highest overall heart rate data, RFT exhibits higher heart rates during the initial phase of the workout when compared to AMRAP. Finally, EMOM stands apart as the one associated with the lowest heart rate values across the entire training program. This outcome is likely attributed to the structured rest periods inherent to EMOM workouts.

**FIGURE 4:**
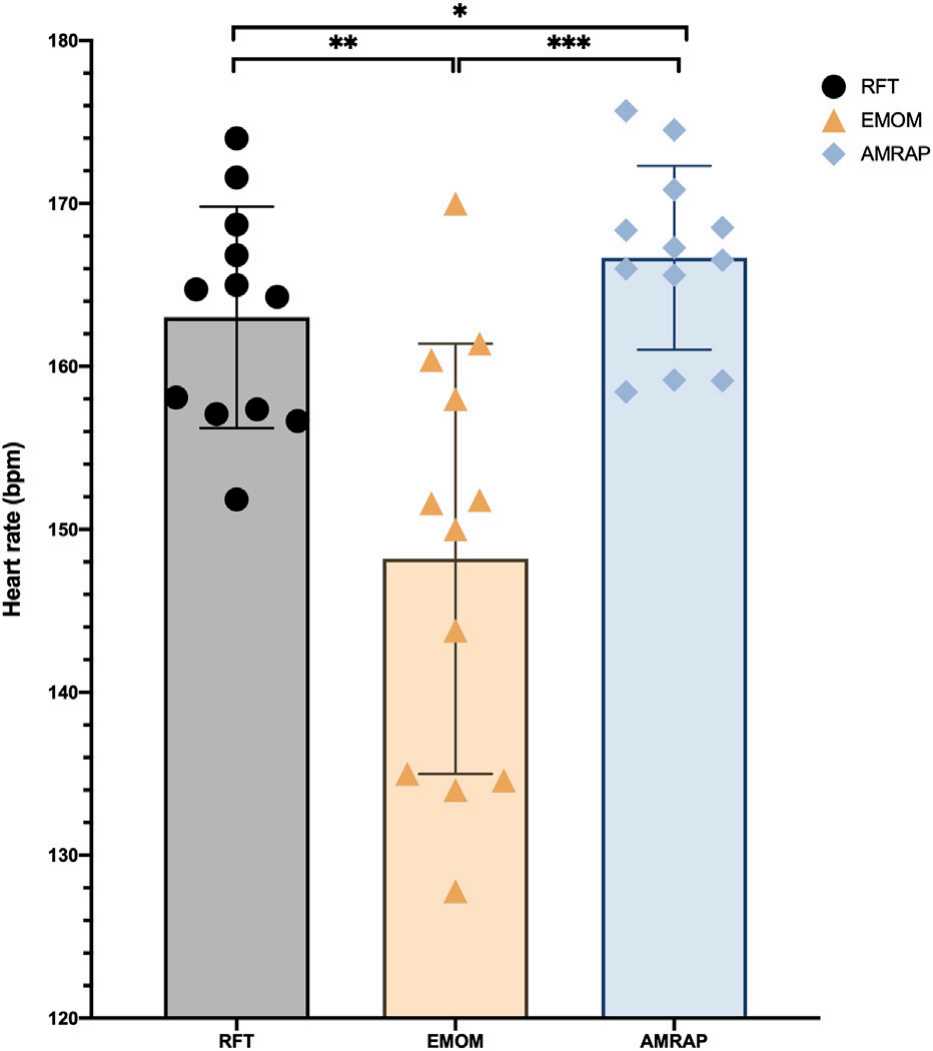
Differences in HR between modalities. Note: * Significant differences for α < 0.05; ** Significant differences for α < 0.01; *** Significant differences for α < 0.001.

Significant differences in heart rate data exist among all exercise modalities (Figure 4). These differences encompass lower heart rate readings in RFT compared to AMRAP (*p* < 0.05), lower heart rates in EMOM when contrasted with RFT (*p* < 0.01), and the lowest heart rates found in EMOM in comparison to AMRAP (*p* < 0.001).

**FIGURE 5:**
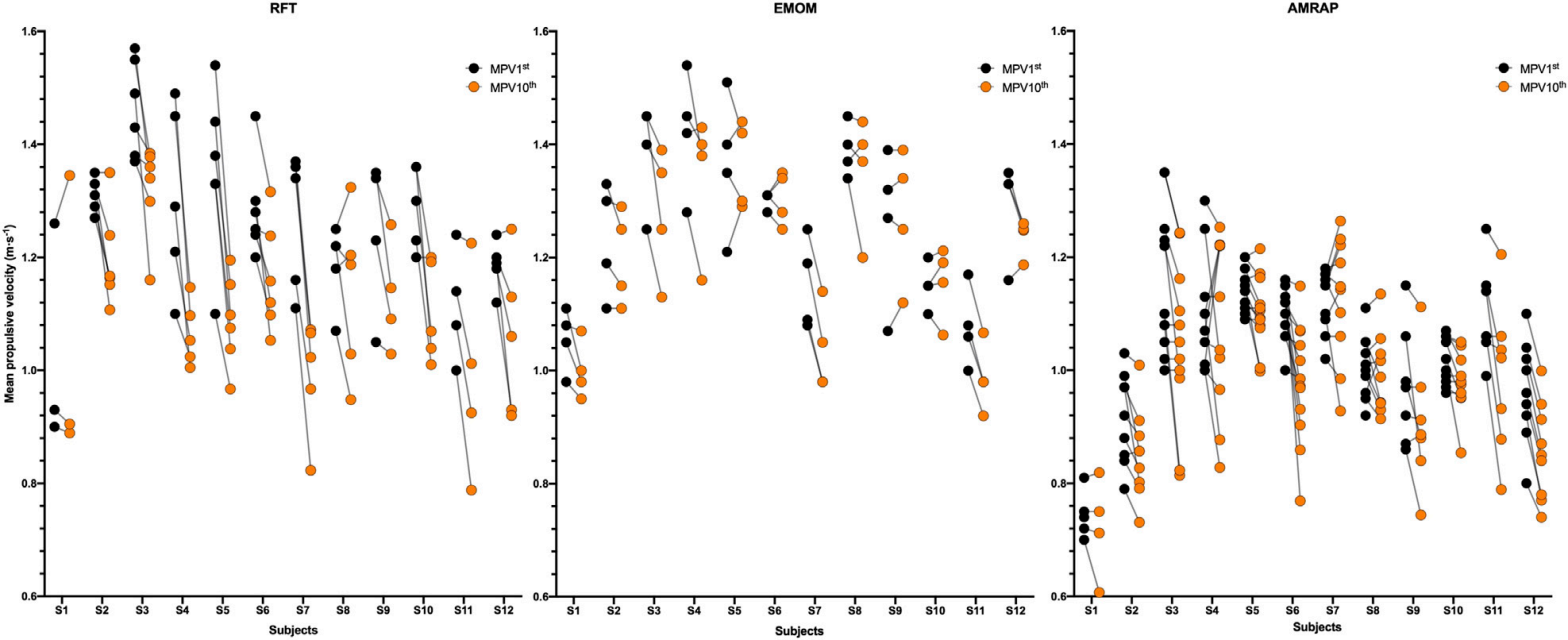
Descriptive data for each subject with the mean propulsive velocity about the first and last repetition in each set according to the modality. Note: MPV1^st^: mean propulsive velocity of the first repetition; MPV10^th^: mean propulsive velocity of the last repetition.

Finally, the descriptive findings comparing repetitions with the highest and lowest mean propulsive velocity (MPV) reveal distinct patterns among the exercise modalities (Figure 5). In both, RFT and EMOM, individuals tend to execute repetitions with notably elevated MPV1^st^ values and exhibit more pronounced discrepancies between MPV1^st^ and MPV10^th^ when compared to AMRAP modality. Conversely, within the AMRAP program, a consistent pattern emerges as participants maintain a relatively stable relationship between repetitions and sets throughout the entire training session.

## 4 Discussion

The aim of this study was to analyse the acute effects on heart rate, propulsive velocity and intra and inter-set velocity loss in each of the “Cross” modalities (AMRAP, RFT and EMOM).

As shown in the introduction mean propulsive velocity (MPV) was considered as the most accurate variable to assess the intensity of strength exercises (Sanchez-Medina et al., 2010). The results present in this research showed AMRAP as the modality with the lowest MPV values. This is due to the fact that it is the method with the longest duration, so the velocity is always lower as the subjects self-regulate, applying less force according to the absolute load (which is possible in a greater extent in experienced subjects with higher levels of strength), to achieve the best performance and maintain the technique of the movement (De-Oliveira et al., 2021). However, no differences were observed between RFT and EMOM. This fact indicates that the athletes can self-regulate the intensity during the modality depending mainly on the characteristics, total duration, and rest periods. This could point out how these modalities cannot be considered as high-intensity modalities given that in high-intensity exercises, if the relative intensity in each exercise were very high, the ability to self-manage the velocity throughout the repetitions would be very limited and conditioned (Sanchez-Medina et al., 2010; González-Badillo et al., 2017). This fact is in contrast with other research on the field (Feito et al., 2018; Feito et al., 2019) and the American College Sports Medicine (ACSM) annual survey in which CrossFit was indicated as the primary reason HIIT workouts (Thompson, 2013; 2017).

The propulsive velocity assessment can provide a measurement of the relative intensity during these kinds of programs, however the fatigue should be analysed with the velocity loss inter or intra sets (Sanchez-Medina and González-Badillo, 2011). Several studies claim that there are high correlations between muscular fatigue measurements and support the use of velocity loss to quantify peripheral fatigue during training (Sanchez-Medina and González-Badillo, 2011; González-Badillo et al., 2017). The analysis of intra-set velocity loss in “Cross” modalities reveals that RFT exhibits the most significant reductions in velocity, particularly during the initial repetition within each set. This observation can be attributed to the strategy employed by athletes who priorities completing the repetitions as quickly as possible, resulting in heightened fatigue and, consequently, more pronounced velocity declines. Notably, these findings contrast with the EMOM program, wherein athletes consistently maintain similar velocities for all repetitions, likely due to the extended rest periods that facilitate recovery between efforts, thereby mitigating fatigue. In the case of AMRAP, velocity loss is less pronounced compared to RFT but higher than that seen in EMOM. This discrepancy may be linked to the pacing and self-regulation strategies employed throughout the entire program. However, it is worth noting that no discernible differences in inter-set velocity loss were identified across these modalities.

The velocity in which the athletes perform each modality and the velocity loss in each set are directly related with the “pacing” established by each participant. In this sense, several studies have recognised different patterns in the pacing strategies during these modalities (De-Oliveira et al., 2021; Mangine et al., 2021). Athletes can self-manage the force applied to the absolute load proposed in the exercise in each series, voluntarily decreasing it. This strategy can be adopted when the absolute loads are moderate-low and will be more feasible in athletes that can achieve strength values higher and thus, lower relative values of the absolute load proposed. The self-management of intra-set recovery can also be deemed, including small recovery times between repetitions or sets of repetitions (often organized as “clusters”) (De-Oliveira et al., 2021; Mangine et al., 2021). Considering these observed trends and drawing from our research findings, it appears that the AMRAP modality demonstrates a distinctive pattern in which athletes deliberately control their applied force, resulting in a pronounced “stable pacing” pattern marked by a consistently stable relationship between intra-set and inter-set velocities. Conversely, particularly in the case of RFT, athletes strive to execute repetitions as rapidly as feasible, leading to a discernible “all/out positive” pacing pattern. These features in exercise management appear to be inherent factors linked to the specific volume and rest structure characteristic of each configuration.

Finally, the response of each modality in the HR noted that the AMRAP presents greater cardiovascular stress with higher HR than RFT and EMOM, surely due to the cumulative effects of the series and repetitions over time. Nevertheless, in RFT the HR is higher in the first steps of the exercise program due to the higher intensity and fatigue. This observation suggests that non-rest routines like RFT and AMRAP exhibit comparable HR values, regardless of variations in volume (duration). This aligns with findings from prior studies indicating that routines with differing volumes tend to yield similar HR values (Fernández et al., 2015; Kliszczewicz et al., 2018). Therefore, in EMOM the heart rate is lower than for the previous configurations, due to the “forced” breaks between exercises (Tibana et al., 2018). Despite EMOM and RFT having similar volumes, the greater intensity in RFT and the lack of rest during the routine appear to be the primary factors contributing to these distinctions. Furthermore, the variances in HR between EMOM and AMRAP could be ascribed to the higher volume and absence of rest periods in AMRAP.

On a more practical level, coaches and athletes must consider the disparities among the “Cross” modalities concerning intensity, fatigue, and pacing as indicated by the research results. These findings should serve as a pivotal reference point for coaches when devising training tailored to the adaptation and the acute effects generated by each training session, contingent upon the chosen modality. It is crucial that the adaptations and effects resulting from training are aligned with the precise goals of the training program or the specific phase within the season.

## 5 Conclusion

This study provides a comprehensive examination about the acute effects in MPV, heart rate, and MPV loss within “Cross” modalities. The research consistently highlights the AMRAP modality as having the lowest MPV values, primarily stemming from its prolonged duration, which encourages athletes to self-regulate their tempo to maximise performance and technique. However, RFT modality exhibits greater fatigue than other modalities, marked by higher intra-set MPV losses. Pacing strategies showed that AMRAP stands out as a modality where athletes effectively reduce applied force, emphasising a “stable pacing” pattern in contrast with RFT in which it can be observed a “all/out positive pacing.” Overall, this research sheds light on the interplay between propulsive velocity, intensity, fatigue and pacing in different “Cross” modalities, offering valuable insights for athletes and trainers aiming to optimise their exercise programs.

## Data Availability

The raw data supporting the conclusion of this article will be made available by the authors, without undue reservation.
